# Women as Agents of Change: Exploring Women Leaders’ Resistance and Shaping of Gender Ideologies in Pakistan

**DOI:** 10.3389/fpsyg.2022.800334

**Published:** 2022-04-06

**Authors:** Nabiha Chaudhary, Anjali Dutt

**Affiliations:** Psychology Department, University of Cincinnati, Cincinnati, OH, United States

**Keywords:** women, workplace, family, gender ideology, Global South

## Abstract

Despite a growing focus on processes to promote gender equity, women remain significantly underrepresented in leadership positions in the Global South. In the present study we focus on the role of familial experiences in shaping and contesting gender ideologies of Pakistani women in the workplace. We specifically examine the reciprocal ways in which women leaders and their family members shape each other’s gender ideologies regarding the workplace. Data collected and analyzed for this study were semi-structured interviews with eight women in positions of leadership in Lahore, Pakistan, and interviews with one family members of each of the women leaders (thus 16 interviews total). Using thematic narrative analysis, we identified three thematic phases: learning gender expectations, resistance, and familial transformation. These phases reflect the progression of developing, resisting, and influencing individual and familial gender ideologies. We document the manifestation of these phases in three specific domains: education, marriage and motherhood, and the workplace. We then discuss how these findings contribute to understanding the experiences of women leaders and perceptions of their family members regarding women’s role in the workplace. Findings from our research provide novel insights into the ways globalization and capitalism continue to shape the socio-cultural context for women leaders in the Global South.

## Introduction

Although women around the world have experienced some increases in leadership opportunities, there remain significant gaps in women’s representation in executive and leadership positions (United Nations Sustainable Development Goals [UNSDG], 2017). To date, the bulk of existing research on women and leadership is conducted in Western, educated, industrialized, rich, and democratic (WEIRD) societies (Henrich et al., 2010). There is substantial need for researchers to document women’s experiences in contexts like Pakistan, where there are likely different barriers women must overcome to attain leadership positions. Transnational feminists’ assert that researchers interested in understanding and addressing gender inequities must attend to the intersecting influences of factors such as race, class, ethnicity, sexuality, nationality, economic exploitation, and other forms of social hierarchy and power that shape people’s experiences and worldviews related to gender (Crenshaw, 1991; Cole, 2009; Grabe and Else-Quest, 2012). To understand processes that support women’s leadership, attention must be given to the intersecting ways in which history, social context, and individual identity shape the unique experiences and opportunities available to women in specific regions (Harding, 2004; Pio, 2019).

One way to gain deeper insight into the contexts that shape women’s lives is through exploration of their familial interactions and relationships. Family members, especially in societies like Pakistan that focus on maintaining strong familial bonds, influence each other’s gender ideologies, including ideas about women’s roles in the workplace (Markus and Kitayama, 1994). Furthermore, exploration of family relationships can provide rich insight into the ways community and societal ideological contexts are internalized and endorsed, ignored, and/or refuted within relationships. Research conducted from a Western lens on women leaders is typically reflective of a neoliberal individualist cultural orientation that values independence and often lacks examination of familial context (Christopher et al., 2014; Marecek and Christopher, 2018). Although studies document that parental support influences young women’s career aspirations across cultures (Li and Kerpelman, 2007; Sovet and Metz, 2014), far less is known about the specific ways in which women and their family members shape and contest gender ideologies in regions where women’s work outside the home is often discouraged. The current study focusses on the reciprocal ways in which women leaders and their family members shape each other’s gender ideologies regarding women’s roles and the workplace in Lahore, Pakistan.

### Gender Ideology in Context

Gender ideology refers to normative beliefs about appropriate or acceptable roles for, and the fundamental nature of, women and men in human societies (Phillips, 2006). In other words, based on the culture one resides, different roles, expectations, and attitudes are associated with being a woman or a man (Wood and Eagly, 2015). Psychological research on gender ideology is conceptualized on a conservative to progressive continuum where beliefs about appropriate roles for women and men vary from traditional to egalitarian (Korabik et al., 2008). Although there exists great variability within and across cultures, societies hold different forms of traditional gender ideologies likely rooted in different historical subsistence and economic systems. Broadly, however, patriarchal values operate on the segregation of spheres demarcating men’s sphere as public, and women’s sphere as private (Sugarman and Frankel, 1996; Ullah et al., 2018). Within this perspective, men are responsible for financial earning and management within the family, and subsequently hold an economically and socially superior position to women who are relegated to be nurturers and homemakers within the family. Such segregation of spheres and roles creates a power differential in which women hold a place of subordination and economic dependence on male family members (e.g., father, husband, or brother) by performing unpaid domestic labor (Leschyshyn and Minnotte, 2014). In contrast, *egalitarian* gender ideology suggests that roles and spheres should not be segregated by gender, and that men and women should have the ability to participate in both outside work and family domains (Kroska, 2007). Undeniably, there exists considerable multidimensionality within each sphere; both political influence and care labor can occur in the private and public spheres. In general, gender ideologies outline roles that shape cultural norms and expectations regarding gender, and are reflected in individuals’ beliefs and behaviors in the contexts of family and the workplace.

The family domain provides a window into understanding cultural ideologies regarding gender that exist broadly at a societal level, and in relations to global structures. Culturally expected norms and gender ideologies are internalized by individuals and are reflected in interactions that occur within the context of family. Additionally, internalized cultural beliefs are often reinforced and/or contested within familial relations that impact beliefs about women in the workplace. For example, previous research illustrates that children acquire their parents’ values, beliefs, and attitudes regarding gender through socialization (Inkeles and Sills, 1968; Carlson and Knoester, 2011). Research in the U.S. regarding intergenerational transmission of gender ideologies shows that parents’ gender ideologies predict their children’s gender attitudes and beliefs (Sutfin et al., 2008; Halpern and Perry-Jenkins, 2016). One study revealed that mothers who complete formal education and hold egalitarian values are likely to have more gender egalitarian children (Ciabattari, 2001). The majority of existing research takes a Western lens and focuses solely on nuclear and single-parent family interactions between parents and their children (Carlson and Knoester, 2011; Halpern and Perry-Jenkins, 2016). Research on the role of siblings and grandparents in shaping gender ideologies is minimal (except in religious context, e.g., Gutierrez et al., 2014). Similarly, little research examines the impact children have in influencing their parents’ gender ideology. Conducting a one-directional study to understand either parents’ or children’s influence in shaping gender beliefs and attitudes does not fully capture the co-creation and co-shaping of gender ideologies. Research is needed that examines culture specific attitudes and beliefs about gender and the role that family members play in transferring, shaping, and challenging gender ideologies (Kroska and Elman, 2009; Kågesten et al., 2016).

In addition to family members influencing the likelihood of women working outside the home, researchers have investigated how women’s labor force participation and equitable job opportunities, in turn, impact the family domain. Research in WEIRD societies reveals that women working outside the home are more likely to negotiate their personal needs and demands, and leave unsatisfactory relationships when they have economic independence (Vespa, 2009; Yu and Lee, 2013). However, WEIRD countries have relatively more gender equity in non-family domains (i.e., economic and education) and more structural and organizational policies related to employment that may provide more support for working women (Davis and Greenstein, 2009). On the contrary, women participating in the labor force in several countries in the Global South often do not have supportive organizational and structural policies, and their labor is often exploited as part of the capitalist and globalized economy (Naples and Desai, 2002). In fact, women working in informal jobs, such as home-based workers lack protection and often do work that is hazardous to their wellbeing (Bonnet et al., 2019). Due to the complexities of these cultural, socio-political, and economic realities, examination of women’s familial context is needed. Family members’ perceptions and worldviews regarding women’s participation in the labor force are shaped by cultural context and can provide insight into how culture filters into an individual’s experience.

### Gender, the Workplace, and Resistance

Ideological beliefs about gender influence women’s opportunities for leadership in workplaces (Eagly and Johnson, 1990; Eagly et al., 2007; Powell, 2012). The segregation of spheres (i.e., public vs. private) perpetuates beliefs and perceptions that women are not suitable for leadership because the workplace is considered a man’s domain and therefore, leadership in workplaces implies a role appropriate solely for men (Garcia-Retamero and López-Zafra, 2006). Moreover, the unequal social status ascribed to the sexes puts women in a subordinated position (Berger and Webster, 2006). Men have more access to the public sphere and therefore more opportunities to participate in overt decision-making processes and lead public conversations that allow them to harness skills valued in the work domain. Simultaneously, women may only get limited access to practice such skills and are expected to prioritize responsivity to interpersonal cues, rather than voicing their own opinions, which can cause women’s leadership skills to be overlooked (Aries, 2006).

Relatedly, researchers have also investigated gender stereotypes linked with leadership that focus on beliefs about the psychological traits of leaders (Schein, 1973, 2001). For example, people are socialized to associate leadership traits such as decisiveness and dominance with men, and therefore rate men as better leaders than women (Booysen and Nkomo, 2010). According to role congruence theory, women leaders face compounded disadvantage because gendered expectations for women contradict with requirements of a leadership role (Eagly et al., 1992; Eagly and Karau, 2002). When women conform to expectations of a leadership role, they are viewed as domineering, competitive, and ambitious (i.e., stereotypical traits of male leaders), and thus perceived as straying too far from expected female gender roles (Rudman and Glick, 2001). Women leaders are expected to choose between roles associated with leadership and roles associated with their gender, and they are considered less feminine when performing leadership roles. The segregation of spheres delineating distinct gender roles for men and women reinforces the notions of leadership as solely for men that consequently limits women’s leadership opportunities. In order to create change toward equitable opportunities for women in leadership positions, it is necessary to examine the ways women leaders challenge and reshape predominant gender ideologies in both public and private spheres.

Although the family and workplace may appear as distinct domains, they are interconnected in the ways they influence women’s lives and prospects (Segalo and Fine, 2020). For example, in the Global South formal education is a precondition for acquisition of successful careers, and a woman’s access to education depends significantly on the ideologies (and economic conditions) of her family (parents and siblings) regarding women’s education. Additionally, previous research shows that change in women’s educational and/or economic participation can create shifts in broader social structures, norms and ideologies (Lefgren and McIntyre, 2006; Dhanaraj and Mahambare, 2019). Dutt and Grabe (2017) conducted a study with Maasai women in Tanzania that examined the ways women challenged traditional gender ideology through involvement in a grassroots education program. Women’s awareness regarding their rights facilitated changes in their ideological beliefs about opportunities and appropriate roles that resulted in women’s political participation and overall ideological transformation. Indeed, researchers have stressed that sustainable change toward equitable realities for women should include transformations in both the public and private spheres (Grabe, 2010, 2017).

Although predominant gender ideologies serve as obstacles to broader participation, they are actively rejected and contested by women in their daily lives (Lind, 2005). Resistance against oppressive structures, systems, and ideologies is studied and documented under psychological research on activism and social movements (Stewart et al., 2011; Dutt and Grabe, 2014, 2019; Chaudhary and Dutt, 2021). However, many women resist inequitable gender ideologies and oppressive systems on a daily basis in their lives without formally labeling themselves as activists or participating in advocacy, and their struggles remain unacknowledged and undocumented. For example, research conducted in Pakistan illustrates that even in post-conflict, oppressive contexts (i.e., structural violence in Karachi and war of terror in Swat Valley), where women’s agency remains constrained by their socio-political, cultural, and economic circumstances, local women exhibit critical awareness of the ways in which their lives are shaped by structures of power, and find creative ways to resist them (Chaudhry and Bertram, 2009; Chaudhry, 2015).

### Context of Pakistan

Pakistan, a formerly British colony, is an overwhelmingly Muslim-majority country with Islam as its official state religion (Haub and Kaneda, 2014). Pakistan is recognized as a conservative country with a patriarchal system reflected in local traditions and cultural values that strongly influence gender dynamics (Critelli, 2010; Cohen, 2011). Patriarchal systems sustain their power and control over the lives and choices of women through institutionalizing segregation of spheres, strict differentiation of gender roles, and restrictive gender norms, such as linking family honor to female virtue (Moghadam, 1992; Winkvist and Akhtar, 2000). Strongly tied to the segregation of spheres and culture of honor is the notion and practice of *purdah* that literally means curtain, and frequently refers to physical veiling of women, and signifies confinement of women in the private domain (i.e., the home; Amin, 1995). Through the idea of purdah, women’s ability to travel alone, participate in the male-dominated labor sectors, and overall mobility is restricted (Mumtaz and Salway, 2005). However, there are other historical, social, religious, political, and regional factors influenced by colonialism and exacerbated by globalization that perpetuate patriarchy and restrictive gender norms for women in Pakistani society (Rai et al., 2007; Mohsin and Syed, 2020). For instance, aspects like socio-economic class, ethnicity, and place of residence of women result in varying gender expectations and gendered experiences (Donnan, 1997). Middle-class women in urban areas of Pakistan are more likely to have careers, work outside the home, and be mobile (Sathar and Kazi, 1997). Thus, Pakistani women and their experiences of gender are not homogenous; they continue to negotiate and redefine their gender roles and social relations (Mumtaz et al., 2003; Mumtaz and Salway, 2005).

Although the overall job market stays predominantly male-dominated in Pakistan, within the last two decades, women’s participation in the job sector has increased and Pakistani women have entered various job sectors, such as academia, banking, medicine, law, engineering, and development. Job sectors, such as academia and medicine, are typically considered more suitable for women. Consistent with the notions of purdah and segregation by gender, it is acceptable for women to teach, preferably other women, and for women doctors to see women patients. Although, women make up a substantial portion of universities and medical school (about 62%), traditional gender ideology and cultural demands lead to the expectation that women prioritize household and family, which results in considerable attrition in higher education (Mohsin and Syed, 2020). Research suggests that while women make up 80 percent of medical graduate students, only half of them become registered to legally practice medicine and even fewer practice medicine in the following years (Zakaria, 2013). Women do continue to excel in the teaching profession at all levels, including higher education, due to the perception of manageable working hours, and the possibility for women to be home before sunset, which allows for household management.

An important factor for women’s attrition in job sectors is the influence of family members in making decisions for the women regarding their marriage, education, and career (Asian Development Bank, 2016). Pakistan’s familial culture is close-knit with an emphasis on communality as a social ethic. The joint family system that entails women living with their in-laws (i.e., parents and siblings of husband) is a structural manifestation of the sense of communality characteristic to Pakistani culture (Mumtaz and Salway, 2009). The prevalent patriarchal system allows men in the household (i.e., fathers, brothers, husbands, and sons) the right to decide the mobility of women that therefore determines women’s access to education and career prospects. The extent to which women are allowed to avail opportunities regarding education, choice of career, and continuity of work varies across a family’s socioeconomic, ethnic, and educational background. Furthermore, while the patriarchal system maintains men’s dominance and authority in the house, the sense of interconnection among family members allows space for negotiation of gender norms and access to opportunities for women. Importantly, women are the key nurturers of social relationships within nuclear and extended families, and thus their socially embedded positions in the family leverages them possibilities to shape their family members’ ideologies through relationships.

### The Present Study

Although there is research on barriers to leadership faced by women in terms of their race, culture, and social norms (Chin, 2010; Johns, 2013; Rosette et al., 2016), less is known about the role of family in shaping subjective experiences of women in the workplace. To our knowledge, no study has explicitly examined experiences of women leaders and of their family members in shaping and contesting gender ideologies regarding the workplace. Thus, in the current study we examine the reciprocal ways in which women leaders and their family members shape each other’s gender ideologies regarding the workplace. In particular, we ask how women leaders’ resist predominant perceptions and gender norms regarding women in leadership positions. Additionally, we examine how women leaders’ involvement in leadership positions impact the gender ideologies of their family members.

## Materials and Methods

### Participants

Eight dyads completed interviews for the current study: eight women in positions of leadership, and eight family members—one member each of the women leaders interviewed. Our sample spanned a wide age range: the age of the women leaders interviewed ranged between 30 and 73, and family members’ ages ranged between 22 and 70. Six of the family members interviewed were men (i.e., a brother, father, and a husband) and two women (a daughter and a mother). The women interviewed held leadership positions in various industries, including non-profit organizations, a private software company, print media, a training institute, and civil services. The range of years in which women have been in leadership positions spanned between 18 months and 32 years. All names provided are pseudonyms, and demographic information such as the names of organizational affiliations and institutes are obscured to ensure the anonymity of participants.

### Research Team and Positionality

This study was conceptualized and designed by the two study authors. The interviews were conducted by the first author who is a Pakistani immigrant doctoral student residing in the U.S. She identifies as a woman, is native to Lahore, Pakistan and is intimately familiar with the socio-cultural context of the region. The second author is a bi-racial Indian-American woman, and faculty member who studies topics related to gendered inequities in the context of globalization. Additionally, two undergraduate students assisted with coding the data. Both are Indian-American, identify as women, are familiar with Indian and Pakistani cultural norms, and were recruited to help with this research because of their interests in psychology and gender. The first author conducted and coded the interviews, and acknowledges the positionality of being an insider and an outsider—a Pakistani immigrant woman, residing in the U.S., and studying her community (Minkler et al., 2002). We are cognizant of the first author’s position (as a Woman of Color, a researcher in community and feminist psychology, and conducting research on her community while she resides in the United States), and how that likely shaped the interviews and coding categories (Braun and Clarke, 2006). Although research shows various challenges associated with being an insider/outsider researcher pertaining (but not limited) to issues of power dynamics and historical trauma (Minkler, 2004), there are several benefits of being an insider as well. The noteworthy, positive features of being an insider/outsider are having access to communities that are traditionally reserved with non-native scholars, cultural consciousness, linguistic proficiency, and sharing similar socio-political histories (i.e., colonized countries; Root, 1996; Anzaldua, 1999; Merriam et al., 2001; Wilkinson and Kitzinger, 2013). Additionally, the second author is an outsider to the specific population of focus in the current study, but her identities as a U.S. citizen and the daughter of an Indian immigrant are laden with power differentials that could impact perceptions of the data focused upon in this study. Utilizing reflexivity as a concept and a process (Dowling, 2006), both authors have taken deliberate measures to be self-aware of our researchers’ positionality and its likely impact on our research practice (Parahoo, 2006; Hesse-Biber, 2007; Ackerly and True, 2010). For example, the authors would meet frequently to discuss each transcript and discuss their codes to find convergence and reach consensus. Additionally, the authors and the coding team bracketed their assumptions at the beginning of the data analysis as an attempt to achieve trustworthiness (Morrow, 2005).

### Procedure

#### Recruitment

This study was conducted in collaboration with a leadership training organization located in Lahore, Pakistan. The collaborator in Lahore solicited recommendations of women holding leadership positions to contact for the interviews. The collaborator asked for recommendations from women participating in leadership trainings at their organizations, working in several different sectors, such as local non-profits and private companies. Eligibility criteria included women who were in leadership positions for at least 1 year and their willingness to refer at least one of their family members to participate in additional interviews. Twenty women were recommended, all of whom were contacted for interviews. Thirteen of the women responded and eight women agreed to be interviewed, along with one of their family members (*n* = 16). All women leader chose to nominate a family member who they trusted and were conveniently available for the interview. Some of the family interviews were conducted with members of the family of origin (i.e., mother, father, and sibling), while others were conducted with members of the current family (husband, daughter, and son). All participants were compensated either monetarily or through culturally appropriate formality gifts of equal value (e.g., fruits, dry-fruits, sweets) when they preferred not to accept money. Five women were not interested in participating because they were not comfortable with providing access to their family members, reflective of Pakistani familial culture values around privacy.

#### Interviews

The semi-structured interview protocol for women leaders focused on specific challenges women faced while attaining and maintaining leader positions, sources of resistance, and their perceptions on how their leadership role impacts their family and community. The interviews with family members focused on perceptions about women in leadership roles and their beliefs about gender roles more broadly (interview protocol in Appendix). Interviews were conducted as per the ease of participants, typically at their homes, workplaces or in public libraries. As part of the consent process, interviewees were reminded that they had the right stop the recording at any time and/or withdraw from the study. The interviews lasted between 40 and 90 min, and were audio recorded. Interviews were conducted in a combination of English, Urdu, and Punjabi language. The participants switched languages as per their comfort and preference within each interview. All of the interview recordings were transcribed and translated into English by the first author for analysis.

### Data Analysis

We used thematic narrative analysis to examine the ways in which women leaders resisted perceptions and gender norms to re-shape gender ideologies regarding women leaders (Marecek and Magnusson, 2018). Specifically, we utilized a combination of deductive and inductive thematic narrative analyses to identify how (a) women leaders’ gender ideologies were shaped by their families, (b) how and why women leaders resisted predominant perceptions and gender norms regarding women in leadership positions, and (c) how their involvement in leadership positions impacted gender ideologies of their family members. Our analyses of the interviews were primarily aimed at identifying recurring themes within their narratives that facilitated understanding of the ways gender ideologies were shaped and contested to bring change in their families’ perceptions of women leaders.

Before formally conducting our analyses, both authors read, reread, and met recurrently to discuss the interviews and form an overarching understanding of their narratives. During this process, attention was given to predominant, as well as contrasting patterns, that consequently resulted in an in-depth framework to account for the discrepancies in participants’ narratives (Josselson and Lieblich, 2001). Next, we focused on a thematic narrative analysis of the interviews to identify recurrent patterns across the interviews, focusing on the ways women leaders and their families were shaping each other’s gender ideologies, and resisting the gendered perceptions of women in leadership positions (Lieblich et al., 1998; Braun and Clarke, 2006; Riessman, 2008). Through this process, we identified three themes, reflecting the progression of resistance and the changes it brought in gender ideologies that addressed the research questions: (i) learning gender expectations, (ii) resistance, (iii) familial transformation. The first two themes, learning gender expectations and resistance are drawn from women leaders’ interviews while the final theme, familial transformation, is exclusively drawn from the interviews conducted with family members. Then, the first author and two trained undergraduate students coded the interviews according to the three themes through a process of consensus coding (Ahrens and Chapman, 2006). The first author and the undergraduate research assistants first coded each of the interviews independently, and then compared results in a collective meeting. Disagreements regarding coding were discussed until consensus was reached. Subsequently, both authors further scrutinized the coded data with attention to the research question. We assessed the validity through continuing conversation about our interpretation of these themes and how they were reflected in each individual participant’s story (Mishler, 1990; Madill et al., 2000).

## Results

Our findings provide insights into the various ways that women leaders’ gender ideologies are shaped by their family members throughout their lives. Additionally, our findings document that women leaders both actively resist the gender ideologies they were taught as youth, and later influence family members’ gender ideologies regarding the workplace. Table 1 provides demographic information about each of the women and their family members.

**TABLE 1:** Demographic information of the participants.

Woman in leadership	Industry	Age	Education	Family member	Relation with the woman	Family member’s age
Aliya	Civil services	35	Postgraduate	Nasir	Father	70
Haleema	Civil services	30	Postgraduate	Tariq	Brother	25
Rabia	NGO	30	Postgraduate	Azra	Mother	58
Fareeha	NGO.	73	Postgraduate	Saad	Son	42
Shameem	NGO	45	Postgraduate	Ali	Husband	48
Neelum	Journalism	68	Postgraduate	Asiya	Daughter	33
Samreen	Trainer and administrator	49	Postgraduate	Usman	Son	22
Bano	Public dealing officer; Private software house	34	Postgraduate	Hammad	Husband	36

The findings are divided into three phases, reflecting the progression of developing, resisting, and influencing individual and familial gender ideologies. The three phases are (a) learning gender expectations, (b) resistance, and (c) familial transformation. We document the manifestation of these phases in three specific domains: education, marriage and motherhood, and the workplace. The phases reflect the process of how women learn gender expectations, resist gender norms and create shifts in their family members’ perceptions of women in the workplace. These phases manifest differently in each domain because each woman’s story is unique and does not follow the same course and/or a linear process. Nonetheless, these domains capture fundamental components that are commonly reflected across women leaders’ narratives of resistance and the shifts it brought in family members’ gender ideologies. Table 2 provides an overview of the phases and the domains as well as coding definitions and illustrative examples from the interviews.

**TABLE 2:** Summary of themes and domains, definitions, and examples.

Domains	Phase 1: Learning gender expectation	Phase 2: Resistance	Phase 3: Familial transformation
*Education*	The gender norms and expectations that women were raised into regarding education	The ways in which women resisted against traditional ideologies to pursue education	The ways women’s resistance resulted in influencing ideologies of family members’ regarding women’s education
*Example*	“My father used to tell me every now and then that I am his son and not his daughter. And he literally used to treat me like a son. I was allowed to do everything that my brother was allowed. He valued education a lot and he sent me to an expensive, private school so I get quality education.” (Samreen)	“My parents arranged my marriage early. I did my masters after I got married because my mother knew that If I started my masters or any job, I would be hard to control. I was the rebellious kind. I told my husband soon after our marriage that I want to get a master’s degree. At that time, it was wise for me to not openly fight my parents and instead have my husband in my corner. My husband was initially reluctant but I told him I don’t want to have kids unless I get my masters.” (Neelum)	“When I think of your research topic of agents of change, it makes me think that It was literally because of me that my family got oriented toward education. The same bother who had torn apart my books and application for college, his own daughter is doing a Masters today and he calls me for advise in everything.” (Shameem)
Marriage	The messages women received as youth about gender norms and expectations regarding marriage, motherhood and an overall marital life.	The ways in which women resisted gender roles and expectations associated with being a wife and a mother through their involvement in the workplace.	The ways women’s resistance through involvement in leadership positions shifted gender ideologies of their family members.
*Example*	“Growing up I was very tom-boyish and my mother would always scold me and tell me to act like a girl. She used to say that even if I become a doctor [of medicine] and don’t know how to cook and keep my husband happy, I will be a failure. There was so much emphasis on learning to become a good wife and a good mother.” (Fareeha)	“As women, we are expected to manage every sphere—home, kids, work, families, everything. I resumed my job when my daughter was 3 months old and my in-laws used to constantly make me feel guilty. But I didn’t quit my job. I used to leave my daughter sometimes with my mother, sometimes my sister would babysit, but I managed. I was constantly being judged as a mother because I didn’t quit my job.” (Aliya)	“Seeing my sister continue working after her marriage, I’d like for my wife to work. I would not force her. It will be her choice to be a housewife or be a working woman at the end of the day. I know women spend so much time, energy and money in earning a degree, I don’t want her to waste it just because she is a woman and it is her job to stay at home and do the things associated with being a female—like child rearing and house-keeping.” (Tariq—Haleema’s brother)
Workplace	The messages women received about gender norms and expectations regarding career choices and appropriate working environment for women	The ways in which women resisted against the gendered perceptions and norms regarding women in the workplace	Influence of women’s resistance through involvement in leadership positions in shaping progressive gender ideologies of family members’ regarding women in the workplace
*Example*	My dad knew very early on that I never wanted to sit at home and be a housewife. I did not want to be a teacher and my dad supported that. He nurtured my progressive and feminist side by giving me access to books and having intellectual conversations with me since my early teenage years (Haleema)	Being a woman, navigating through bureaucracy, which is a male-dominated context, is very hard. Men in powerful positions harass you in many ways. Just the way they look at you, I tell you, you feel naked. They create hurdles for you and think women cannot make rational decisions. But I prove them wrong. I don’t back down and I do what I find to be a better solution. I stay assertive and open to discussions. And now my male colleagues know that I can work as well as they do. (Rabia)	“I had a positive perspective about working women. I wanted to be like my mother. She was my role model and she instilled her passions and creativity in me too. She would push me to be a part of debates, essay competitions, and be a zealous reader. I started writing short stories for papers at the age of eleven, and I was getting published after that every now and then. I knew that I would either follow my mother’s footsteps and be a journalist, or have some other career. My mother was my role model and wanted to be like her.” (Asiya—Neelum’s daughter)

### Education Domain

Education plays a fundamental role in intellectual growth, securing jobs and successful progression in careers. Topics examined in the education domain center on the ways women leaders made sense of the norms and opportunities for women’s acquisition of education. It also focuses on their processes of resistance against inequitable educational prospects and discourses and freedom to avail those opportunities. Moreover, this domain illustrates the outcomes of women’s resistance reflected in shifts in family members’ gender ideologies regarding women’s education.

#### Learning Gender Expectations

Gender ideologies expressed in families shape and outline foundational ideas about norms and opportunities for women related to education. Through this process of familial socialization, women leaders’ initial perceptions, views, likelihood of accessing education, and eventual chances of pursuing careers were developed. For example, Haleema, a woman working in a leadership position in the civil services shared:

When I look back, I had the basic equality for the basic rights [from parents] that I now realize is rare in our culture. I had equal access to education and freedom to pursue a job, and driving the car etcetera, just like my brothers. My dad has always supported me and I am where I am because of him. He even pushed me to do masters after I had already landed my dream job. (Haleema).

Haleema is describing the way her parents’ socialization entailed providing her with similar opportunities for education and pursuing a career as her brothers. Pakistani parents often encourage their sons to pursue higher education, but not their daughters, in part because cultural norms prescribe sons as responsible for taking care of parents in their old age (Kaul, 2018). Thus, parents tend to invest more in sons’ education that could help them in finding stable and decent jobs. Haleema explained how her parents did not follow this norm and supported her to pursue higher education. She internalized the messages of having equal opportunities regardless of gender. Moreover, she attributes her success to her father’s financial and moral support, and acknowledges that not everyone has this privilege. Familial socialization around equal opportunities for female and male children facilitated her pathway to be a successful leader.

In contrast to Haleema, Shameem’s family held conservative attitudes about women receiving education. Shameem, a leader in an NGO, shared that she applied to college with her elder sister’s help, however, her elder brother was not supportive. She shared:

My brother tore apart the application form and told me strictly that because I am his honor, he cannot let me go out and study in a college because, according to him, “I know what happens in colleges. Nobody studies and girls just indulge in romantic relations and do shameless acts. I won’t let you embarrass me in front of the world and make me ashamed of myself in front of my friends.”…I had to wait several years to pursue college after that because I had to figure out a way. (Shameem).

Shameem’s experiences of not being allowed to pursue college education illustrates her family’s gender attitudes toward women’s acquisition of formal education, and the way it shapes her understanding of the patriarchal system and the inequities this perpetuates. The discourse of women being the honor of the family often hinders women’s freedom to study and work, and delineates their life trajectories in the context of Pakistan (Sev’er and Yurdakul, 2001; Khurshid, 2012). Moreover, this example highlights the differences in support from family members in women’s pursuits of education. Shameem’s brother’s conservative views about women’s access to education influenced her opportunities to receive education, and shaped her initial understanding of inequities regarding women’s access to education. Societal norms and ideologies are internalized, learned and often reproduced without disputing through familial interactions and relationships.

#### Resistance

Resistance refers to the unique ways women leaders defy, challenge and/or modify prevalent unjust gender norms regarding education. As discussed, some interviewees experienced obstacles in acquiring college education due to their family members’ conservative gender ideologies that equated women’s education with loss of honor and family traditions. However, women leaders challenged the unjust norms and contested these oppressive ideologies by seeking education. Returning to Shameem’s interview, she explains the process through which she was able to resist gendered expectations:

After matriculation, my brother did not let me study…Years after, when I joined [name of organization], I was still just a matriculate, and did not have an intermediate degree. After joining the organization and being in the company of so many educated and feminist women, I finally had the motivation and support to pursue my studies… Also, by then, I was financially contributing in my house for years, and I had a say. My contributions gave me space and got my brother and everybody to take me for who I am to some extent. (Shameem).

Here, Shameem explains the course of advancing her education after a gap of several years. Although her brother did not permit her to pursue studies, she started tutoring at home, and from there, she started working with a community organization. After years of working, she joined a new non-profit organization, and found herself surrounded by feminist and educated women who encouraged her to go back to college. Shameem’s process of resistance involved looking for alternatives to continue to grow as a person rather than giving up. When she could not go to college, she started work that provided her with opportunities to be financially solvent and develop agency and autonomy to ultimately advance her studies. For her, the feminist space led to growth and self-actualization. In addition to having motivating and feminist colleagues, Shameem highlights the importance of being financially independent in ensuring a woman’s ability to follow her academic pursuits. She underscores the role her financial solvency played in providing her a space for resistance against her brother and following her academic aspirations. Previous research conducted in the Global South shows similar patterns where women feel more empowered to resist and create change in their lives after gaining financial independence (Grabe et al., 2015). Importantly, Shameem’s struggle and journey of going back to college highlights that resistance is not always explicitly aggressive, immediate and radical. For her, it was strategic and contingent upon the specific realities of her context.

#### Familial Transformation

The final phase illustrates shifts created by women leaders in the gender ideologies of their family members in the domain of education. Witnessing the ways women leaders challenged gender norms regarding women’s opportunities to pursue education and attain leadership positions while navigating male-dominated workplace environments influenced their family members’ perceptions about education. Women leaders’ family members identified shifts toward gender progressive attitudes and overall positive views about women’s access to education. For instance, Shameem’s husband, Ali, shared:

Shameem is a fearless woman. She has been through so much but she uses it as her strength. Her love for education and growing inspires me. And because of her, our children love to study… it is also solely because of her success and love for studying that her nieces are getting educated. Her brother was extremely conservative and against girls getting college level education. But his daughters are doing masters and Shameem is who they look up to. (Ali, Shameem’s husband).

Ali emphasizes the inspirational role Shameem plays in sparking interests for studies in their children, as well as paving a way for her nieces to get education. Furthermore, Ali highlights the progressive shift in Shameem’s brother’s perception toward girls’ and women’s education. Previously, Shameem shared how her brother did not allow her to attend college and she had to wait several years to resume her education. However, Shameem’s resistance and success in creating a name for herself led her brother to learn the importance of education for women and to provide his daughters equitable access to education.

Across the interviews, family members shared that witnessing women in leadership positions had influenced their beliefs about women’s access to education. For instance, although Haleema’s family was already encouraging of her education, seeing her as a successful leader strengthened their perceptions regarding women’s rights to get educated. In a family context where women’s advanced education was initially discouraged, Shameem’s resistance created shifts in her brother’s perceptions of women’s education. Shameem’s success demonstrated that women seeking education do not bring shame, and this opened doors for other women in the family to pursue education, as well.

### Marriage and Motherhood Domain

Marriage and motherhood are typically considered crucial developmental milestones in a woman’s life in Pakistan (Mumtaz et al., 2013). Due to the close-knit culture that revels in the sense of togetherness among families, these milestones are celebrated, encouraged, and perceived as the most important responsibilities for a woman to lead a fulfilling life. Following the expectation for women to prioritize their marital lives, the pursuits of education and career often create difficulties. Familial relationships can foster the internalization, reproduction and/or contestation of these norms. Several interviewees mentioned the ways they were mostly socialized to prioritize their roles as mothers and wives, and rate education and career as secondary. Furthermore, some interviewees received messages at home to not work after marriage and/or having a child, or look for a job that allowed for more time serving the needs of the family.

#### Learning Gender Expectations

An important aspect of being socialized into family’s gender ideologies and views regarding women’s roles include making sense of the decisions elders in the family make for women. For example, Samreen, a senior teacher trainer, shared:

I was good at studies, but my parents decided to marry me off while I was still doing my masters’ degree. It took me 6 years to convince my husband to let me be a working woman. He was from a landlord family where no woman ever worked outside the house in their entire family. After a lot of fights, he gave up and he said I could work. But he had one condition: work in a school only and be back before 3 p.m. (Samreen).

This example highlights the process of learning gender norms and navigating familial perspectives about married women pursuing careers. Samreen’s academic journey was interrupted to get married and she adapted to the decisions made by her parents. Samreen developed an understanding about women’s limited options for pursuing a career after marriage through this experience.

Another important aspect associated with families shaping women leaders’ gender ideologies concerning marriage and motherhood was the ways women internalized gender expectations. Although women receive messages to give precedence to their marital life, some women expressed feeling unfulfilled as homemakers. Several women described experiencing psychological stress due to holding contradictory beliefs about having to choose between work and their individual growth, and satisfying normative gender role expectations of solely being a mother and a housewife (Steiner et al., 2019). For example, Fareeha, a co-founder of a feminist NGO, shared:

My son was around 3 years when I started losing my mind being at home all day long. My mother saw my condition and she helped me out. My mother was extremely feminist and wanted me to prioritize my sanity and made me realize there is more to a woman than her house. She taught me that the house is important, but a woman herself is also important. That’s how I started my job again. (Fareeha).

Previously in her interview, Fareeha shared that after she conceived her son, she quit her job to follow the gender norms of being a devoted housewife and a mother. However, Fareeha soon realized that she was feeling unfulfilled without her job, and that being a home-maker was taking a toll on her psychological health. Fareeha described that it was due to her mother’s push and support that she went back to work. This example illustrates the psychological dilemmas married, working women experience to fulfill the gender role expectations associated with being a mother by sacrificing their sense of self and need for self-actualization. Additionally, this example shows how familial support and family members’ gender ideologies that value women’s wellbeing shape women leaders’ worldviews and resulting choices. Fareeha learned from her mother and internalized the need to foster her passions along with taking care of her son to lead a content and healthy life.

#### Resistance

Although in Pakistan dominant cultural norms, ideologies, and expectations regarding gender hold marriage and household as particularly important for women in general, families reinforce, contest, or negotiate these expectations in unique and varying ways. Since marriage and motherhood are important milestones in Pakistani women’s lives, pursuing a career for the married interviewees brought several challenges. For example, Fareeha described her struggles as a mother starting a non-profit and how she kept going in the face of challenges:

When we started the NGO in 1980s, people were against us. They called us elitist, educated, feminist, etc. I was told many times to go home and just take care of my son. I did my masters after getting divorced in my late 30s. And, I used to often question myself if what I do is worth the name callings. But, I kept going. What I do. What I did. My activism, my career, my passions, they are all worth it. (Fareeha).

Fareeha is reflecting on the backlash she experienced for pursuing a leadership role. She describes how people used to judge her as a bad mother because she was working. She was called names for being part of what was considered the man’s public sphere. She further reflects on the numerous times she had questioned herself if being a woman leader was worth the backlash she faced. However, she resisted and continued her job, activism and passion. This example illustrates the unique challenges that women leaders who are mothers face and the resilience and courage it takes to keep resisting and pursuing one’s career. Fareeha’s resistance is reflective of both personal and professional struggles and injustices faced by women. Fareeha, as an activist and a divorced single-mother during the 1980s in Pakistan was resisting hostility toward assertive and vocal women in the non-profit sector, and against the taboo of being divorced and raising a child on her own.

#### Familial Transformation

Women leaders’ resistance shifted expectations about women’s role as mothers and wives by challenging the gender ideologies regarding marriage and motherhood. Although women leaders’ family members indicated marriage and motherhood as important aspects of a woman’s life, they highlighted that women leaders can be good mothers and wives while pursuing a career. Additionally, interviewees identified how a working mother serves as a role model to her children and other people in the community. Specifically, this theme highlights the ways family members developed progressive attitudes regarding working women’s roles as wives and mothers. Fareeha’s son, Saad, described how his mother shaped his feminist consciousness:

I consider myself pretty progressive. My mom and aunt taught me how to cook and take care of myself since childhood. I love doing household chores. And I do think, how I was brought up and philosophies of my mother have shaped my feminist consciousness. I believe in equity and teamwork. Women should work if they want to work. They have a right to make decisions for themselves… My mother worked all my life and I am proud that she showed me that working women are as good mothers as the ones who stay at home. (Fareeha’s son, Saad).

Here, Saad describes himself as a progressive man who is an advocate for women’s freedom of choice and gender equity. He attributes his feminist consciousness and ability to do chores that are stereotypically associated with women in the context of Pakistan, such as cooking, cleaning and managing the house, to his mother’s life philosophies. He further highlights that his mother served as a role model for him to know that women can pursue a career and be good at fulfilling their responsibilities as mothers and wives. Saad’s awareness of how women are often not allowed to have equitable access to opportunities due to the gender norms in society is shaped by Fareeha’s resistance and modeling of feminist ideas. Indeed, Saad’s gender ideology is reflective of the transformation that continued across generations, transmitted from her grandmother’s and mother’s beliefs about women’s roles.

### The Workplace Domain

The third domain, the work place, highlights the kinds of messages women leaders’ received regarding women’s possible role in the workplace from their family members, how they resisted these views and practices, and how their resistance transformed their family members’ gender ideologies.

#### Learning Gender Expectations

Central to this domain are the different ideologies and perspectives that families held and communicated to women leaders, ranging on a continuum from traditional to progressive norms regarding women’s participation in the workplace. This included culturally appropriate job sectors and environments that encourage women’s participation. In addition to certain jobs being deemed more appropriate and in line with the gender roles assigned to women, the interviewees underscored the visions their families had for their future careers. For example, Aliya shared:

It was my father’s dream that I become a self-sufficient and independent woman. He raised me in a way that it was understood that I will work after my studies are complete. When I completed my studies, there was this teaching job advertisement in the newspaper. He was very motivating. He said, “go ahead and apply.” And I did, and I got the job, and the rest is history. (Aliya).

Here, Aliya is describing that growing up she was encouraged to pursue a career. Her father supported and encouraged her to work, earn, and be an independent and self-reliant woman. In the context of Pakistan, where most women are housewives and financially dependent on their male guardians (i.e., husbands, fathers, or sons) to have their basic needs met, Aliya’s father wanted her to be self-sufficient. Aliya internalized the support and perspectives of her father and from early on she knew that she would have a career. Another interviewee, Haleema shared a similar opinion, *“when a woman is financially solvent, she has a lot more power. You are less submissive.”* Thus, Aliya was raised and socialized to believe that women should work to be self-sufficient and such messages empowered her to progress in her career.

In contrast, another interviewee, Rabia described how her family members discouraged her interests in working outside the home:

I am from a village where women don’t even get educated, let alone work. When I used to go to [high] school, my brother used to accompany me because as a woman I was not allowed to go outside the house alone… My family believes that if I work, I would not get good proposals for marriage. People in our society think that if a woman leaves the house to work, then she is no longer a woman. Another thing is that some jobs are considered okay for women. For example, if I teach, I will not be challenging the norms. People will be able to digest it. But, starting and owning your own organization is considered a manly job. “Maybe that is why my family never wanted me to work.” (Rabia).

As Rabia reflects upon her families’ attitude toward working women, she describes that she comes from a rural background where women do not acquire higher education. In the rural areas, there is often either none or only one higher education institution situated at the outskirts of the town that requires students to travel in the absence of working transportation system. She further explains that growing up, due to her gender, she was not allowed to go to school alone, and therefore realized that she would not be allowed to go to work unaccompanied either. Restrictions regarding women’s mobility are a social norm practiced to maintain the culture of honor and segregation of spheres (Amin, 1995). Rabia describes that certain professions, such as teaching, are considered appropriate for women, as compared to starting an NGO. Her family members believed that she would jeopardize her chances of securing a decent marriage proposal if she worked at a job that would compromise perceptions of her femininity. This example highlights that parents internalize and reinforce gender ideologies and societal expectations to secure their daughters’ future prospects and avoid social stigma.

#### Resistance

Workplaces, typically designed as a domain for men, pose several challenges for women, and thus required resistance and adjustment from women to survive and excel in the workplace. The interviewees identified various challenges along with ways of contesting male centered norms in the workplace related to perceptions of women’s competence, socioeconomic status, and communication and leadership styles. Bano, a leader in a software company, described how her clients doubted her competence because she was a woman:

Most of our business partners and clients are men. When I talk to them, for some reason, they don’t take women seriously. They would always inquire about some male colleague or boss or some other male from my organization that they could interact with instead. It doesn’t matter how capable you are and how good you are at your job. They just have this image of women in their heads that women are incapable of holding meetings and making good decisions… But, I have dealt with different kinds of clients and I don’t back down. I usually ask them “why do you want to talk to my boss? Am I not answering your queries well? Can something be improved?” And then they are just quiet. Then they know I am the one who is going to deal with them and nobody else. (Bano).

Bano is describing the stereotypes that the society holds, especially men, regarding women being incapable of leading, managing, and making decisions in the workplace. She explicates that her clients prefer interacting and discussing their businesses with men because they believe that women are not competent to manage corporate dealings and make sound decisions. The misogynist attitudes of men reflect the broader gender stereotypes held by the society regarding women leaders’ capabilities, and how it creates unfair complications for women leaders. Nonetheless, Bano resisted these attitudes with persistence, confidence, and courage, and stayed dedicated to her career.

Likewise, Haleema described how being a woman created difficulties in communication with subordinates and colleagues, and the ways women leaders have to make conscious efforts to be seen as professionals:

What used to frustrate me the most was the fact that people, my male subordinates actually, they did not see me as a professional and used to try to manipulate me. Like, I remember when I started my job, this subordinate of mine, came to my office with an application to get leave and he was crying with actual tears. So, I inquired, what happened and he handed me over his application. The application was to ask for leave to go for *Hajj* [Islamic pilgrimage]. I got so furious that it was a valid reason to ask for leave, I’d have permitted that even without his drama. He was crying because I was a woman. So I told him to go outside and come talk to me when he has stopped crying and can talk like a normal person. I would have been considerate if it was a real issue. But he was just trying to gain sympathy from me. So you know as a woman you are very conscious of the fact that you are a woman and you need to act certain ways and be professional because people want to use that against you. (Haleema).

In this example, Haleema highlights the challenges she faced as a woman leader. She explains that because women are considered more emotional than men, her subordinate tried to rouse her emotions to gain sympathy to get his leave approved. She also discusses how such behavior reveals that her male subordinates do not think of her as a professional person, but rather as just a woman. She resisted that attitude by being firm and clear about her capabilities as a leader who can make rationally driven decisions. Haleema connects her subordinate’s attempt to use her emotions for his gains with woman leaders’ struggles of navigating professional settings and consciously making efforts to present themselves as professional, and not just emotional beings.

The experiences of gender disparity and women leaders’ resistance to the gender norms in the workplace often occurs across the intersections of their social identities. For instance, while discussing the challenges she and other women leaders face, Rabia shared:

One’s socioeconomic status changes the kind of challenges they face while working. If you belong to a rich, reputed, well-connected family, it will be easier for people around you to take you as a boss or a leader. Because I belong to a weak and poor family, it is hard for my colleagues to take me seriously because they know my family is unsupportive—both financially and emotionally… Women like me have to resist at many levels. (Rabia).

Rabia is describing how economic disparities create several social inequities for women in the workplace (Crenshaw, 1991). She understands that the disadvantages stemming from her lower socioeconomic background create more challenges for her in the workplace. In male-dominated job sectors that favor men as leaders, a woman leader’s lower socioeconomic status places her in positions of disadvantage, and makes it harder for people to take her as a compelling leader. Despite the various intersectional inequities, leaders like Rabia keep persisting and resisting the unfair yet prevalent encumbrances posed by their gender and socioeconomic status.

Overall, this theme illustrates the various ways women leaders resist limiting gender ideologies related to the workplace. Through challenging norms and overcoming cultural and systemic barriers associated with women in the workplace, women leaders persisted in their efforts to push back against the gender ideologies. Moreover, as part of their resistance, women leaders designed strategies to continue the resistance in their specific contexts and unique situations.

#### Familial Transformation

Lastly, women leaders’ contesting and negotiating of gender norms and expectations shaped their family members’ gender ideologies regarding women in the workplace. Only one of the interviewees had a working mother. In fact, most of them were the woman first in their family to pursue higher education and/or leave the house to pursue a career. The women leaders interviewed, however, oriented their family members toward women’s participation in the workplace. Their resistance and success shaped the resulting gender ideologies of their family members. For example, Neelum’s daughter, Asiya shared:

I vicariously learnt to be a leader and a strong headed woman because my mother was one. After my mother become a working lady, my dad’s side of the family gradually not only accepted that but they used to boost off that their daughter-in-law is a successful woman. Since this is the kind of environment I grew up in, I wanted to be like my mother. I have a daughter now and I am still working. I think, in our society, we are all interconnected. If my mother shaped me to be a strong woman, in the same ways, other women working in leadership positions are shaping women around them. And not just women, men as well, because women can shape the mindsets of both men and women because they are so integral to any household. Children always look up to their mothers. (Asiya, Neelum’s daughter).

Asiya reflects that she vicariously learned to be decisive and a leader because her mother was modeling behaviors of a strong, independent woman. Furthermore, Asiya highlights a shift in her mother’s in-law’s perceptions about her mother’s work. Although, initially Neelum’s in-laws were unsupportive of her job, gradually they accepted her desire to work and felt honored to be related to her. Thus, Neelum shaped gender ideologies of her in-laws, as well as her daughter. Additionally, Asiya explains that in Pakistani culture, a mother has the potential to influence the gender ideologies and perspective of people in the family because of her interconnected and respected status in the household. It is through the sense of interconnection within families that women can serve as role models and support each other to create a gender equitable society. Indeed, Asiya’s positive perceptions about women pursuing careers are reflective of the continued transformation happening intergenerationally.

## Discussion

In a globalized world where gender disparity continues to be a pervasive problem, identifying pathways that women leaders in the Global South take to resist and change inequitable outcomes for women is of urgent importance. The current study highlights how women leaders experience intersecting inequities that shape the culture-specific realities related to women’s empowerment in the context of Lahore, Pakistan. Focusing on the central role that familial interactions and relationships play in influencing gender ideologies regarding the workplace, we examined the ways women leaders resist and transform traditional gender ideologies in Pakistan. The findings from the current study contribute to understanding the psychological processes that women leaders employ to resist and shape gender ideologies regarding the workplace, and underscore the important role that familial bonds play in nurturing and contesting gender ideologies in cultures like Pakistan that are close-knit and value interdependence.

Our findings illustrate the co-shaping of gender ideologies between women leaders and their family members. Specifically, through the three identified phases, we illustrate ways women leaders are socialized into understanding gender norms and expectations, resist unjust inequities related to women in the workplace, and through their continuous resistance promote progressive gender ideologies in their families. Although the phases manifest differently in each interviewee’s narrative, across the domains of education, marriage and motherhood, and the workplace we identify psychological processes experienced by women leaders that result in resisting and transforming gender ideologies of their family members. Overall, the findings show how family relationships can provide insight into the unique ways societal ideological contexts are internalized, reproduced, and/or contested within familial relationships.

Our study’s findings reveal the context-specific psychological processes women employ to navigate patriarchal and societal norms and to resist traditional gender ideologies to attain leadership positions in Pakistan. For instance, in line with previous research, our interviewees’ reflected on the importance of women being financially independent because it can increase agency and decision making power among women in Pakistan (Noreen, 2011). Additionally, interviewees referred to the restrictions regarding women’s mobility as a social norm practiced to maintain the culture of honor and segregation of spheres (Amin, 1995). However, it is important to note that cultural norms of honor, segregation of spheres, and expecting women to prioritize household and families sometimes serve various functions that counter structural inequities and oversights. For example, in contexts such as Pakistan where legal institutions and government have not been efficient at legislating and systematically enforcing laws to ensure women’s rights and dignities (Critelli, 2010, 2012), families serve that purpose of protection by increasing women’s surveillance and cultivating a norm for girls and women to be home before sunset. Additionally, the lack of social and legal support for women experiencing workplace harassment, especially in male-dominated fields, increases parents and families’ concern regarding women’s safety. Nonetheless, a study conducted in Pakistan shows that families, especially fathers, play an instrumental role in women’s successful career by providing them academically encouraging childhood environment and achievement motivation (Saleem and Ajmal, 2018). Overall, the current study does not attempt to portray Pakistan as illogically patriarchal, instead it underscores that there are inequitable and detrimental outcomes of families’ internalization and reinforcement of ideologically and structurally gendered discriminatory beliefs and practices to maintain the status quo for women’s empowerment and attainment of leadership positions. Furthermore, Western feminism has the tendency to portray women in the Global South as oppressed and lacking agency (Mohanty, 1984; Kurtiş and Adams, 2015). The current study aims to disrupt such victimizing and universalizing narratives and biases in scholarship.

### Limitations and Future Directions

Although our study’s findings contribute to understanding the role familial relationships play in shaping and contesting gender ideologies regarding women in the workplace, there are some limitations. We interviewed eight women leaders working in non-traditional sectors that are non-representative of their sector, and thus limit generalizability. The goal of the study was not to be generalizable, but rather to deepen understanding of gender ideologies through participants’ experiences. However, we shared the findings in multiple settings (e.g., with coders, and at a conference) to increase interpretive validity of our analysis. Future researchers should target a relatively larger sample with an emphasis on achieving sector-wise saturation to gain deeper insights into the experiences of women leaders in specific sectors. Furthermore, the family members interviewed in this study were selected by the women leaders, and therefore posit a potential limitation of bias and convenience. Nonetheless, considering the cultural norms of reserved interactions with people of opposite gender and of familial privacy, the selection of family members by the women leaders is a culturally sensitive and relatively less intrusive way to learn about their perceptions. Our study focuses on the ways women leaders’ resistance created progressive shifts in their family members’ gender ideologies; however, we acknowledge that this impact was likely not experienced uniformly by every family member. Indeed, family members experienced domain-specific changes regarding their perceptions of women in the workplace, yet change in one domain does not imply change in others. For example, shifts in one’s views about women’s access to education does not mean that they also endorse women’s choice to work after marriage. Additionally, changes in domains where women are increasingly accepted for the work outside the home doesn’t necessarily alleviate any of the expectations that women must excel as mothers, wives, homemakers, daughter-in-laws, etc. As capitalist pressures necessitate that more women enter the workforce, in addition to the desire that many women have to work outside the home, future research should examine the impact that pressure to excel in numerous domains has on women and their communities wellbeing.

Pakistan is a country with diverse languages, cultural norms and practices, and varying resources and opportunities contingent upon the provinces one resides in. It is important to note that the women leaders interviewed in our study all resided in Lahore, the capital of the province of Punjab, and the second largest city in Pakistan and thus have relatively better access to educational and employment opportunities, resources like public transportation, and relatively diverse and broadminded attitude regarding women’s participation in the workforce. Future research should explore women leaders’ experiences in a variety of settings to better understand the ways gender ideologies are shaped and contested. Furthermore, all but one of the women leaders interviewed were married at the time of this study. Future research should explore the experiences of women whose paths regarding marriage and motherhood diverge from traditional expectations, exploring the impact of the decisions they make.

### Implications

The current study highlights how family relationships can provide insight into the ways societal ideologies and structural inequities are internalized and endorsed, disregarded, or contested within relationships. Additionally, this study offers insights into psychological processes employed by women leaders in the Global South to resist prevalent ideological barriers and achieve leadership positions. In light of previous literature and the current study’s findings, it is evident that gender ideologies and familial relationships carry the potential to shape a culture that can empower women in sustainable ways. Thus, understanding the dynamics involved in the internalization and evolution of gender ideologies can be a useful tool in designing and implementing contextually appropriate programs and policies for women’s inclusion in the workplaces.

## Conclusion

Globalization and capitalism continue to shape the socio-cultural context for women in the Global South. In Pakistan, increasing shifts in the demands for labor, and decades of Western endowment in promoting women’s access to education have created a push for women to pursue formal, competitive education and careers. However, such shifts in the spheres of education and labor market have neither translated into better workplace environments for women, nor into the overall socio-cultural and ideological adjustments. Despite a growing number of women getting higher education and joining the workforce, women continue to face sexual harassment at work and an overall hostile work environment, along with ideological barriers to pursue careers. Through the utilization of the family lens, the present study provides novel insights into the ways the structural shifts driven by globalization and capitalism impact women’s households and work-related experiences. The current study, therefore, underscores the ways women experience and resist inequities and discrepancies at the intersections of the interconnected domains of the family and the workplace that shape their lives and prospects.

## Data Availability Statement

The original contributions presented in the study are included in the article/supplementary material, further inquiries can be directed to the corresponding author/s.

## Author Contributions

Both authors listed have made a substantial, direct, and intellectual contribution to the work, and approved it for publication.

## Conflict of Interest

The authors declare that the research was conducted in the absence of any commercial or financial relationships that could be construed as a potential conflict of interest.

## Publisher’s Note

All claims expressed in this article are solely those of the authors and do not necessarily represent those of their affiliated organizations, or those of the publisher, the editors and the reviewers. Any product that may be evaluated in this article, or claim that may be made by its manufacturer, is not guaranteed or endorsed by the publisher.

## Appendix

### Semi-Structured Protocol for Women in Leadership Positions

1. What was growing up like?
2. What are your earliest memories of understanding what it meant to be a girl?
3. Did you have any women in your family who worked outside of the home?
4. What got you interested in pursuing this career?
5. Who were your role models for seeking experience in leadership?
6. Tell me about your work journey? What was your first job/work position?
7. How would you describe your current job/work position?
  a. What kinds of activities do you do during a typical day at your job?
8. How has your current position been rewarding?
  a. What are some of the things that make you feel welcomed and included in this space?
  b. Tell me a story about a time you felt valued or like you had a voice here.
9. What are some specific challenges you faced and/or still face in this job?
  a. What are some of the things that make you feel unwelcome or excluded in this space?
  b. Tell me a story about a time you did not feel valued or like you did not have a voice here.
10. How do you cope with these challenges?
11. What are some of the biggest strengths and resources you have?
  a. Does your involvement at [name of organization] affect your strengths and how you think about those strengths? If so, how?
12. Can you tell me some of the issues you face as a woman in your current leadership position/industry? and how do you think they should be addressed?
13. In your view, what sort of challenges do other women in leadership positions face in Pakistan?
14. What does is look like for women today as they are trying to achieve work life balance?
15. In your view, what opinions do other people have about you, since you hold this position of leadership? Among family and extended relationships?
16. Have you noticed if people treat you differently now that you hold this leadership position? How so?
17. Do you think being a working woman and in leadership position has any influence on your family? (parents, husbands, in-laws, children, friends, extended family etc.) In what ways?
18. In your view, how has the Pakistani society changed in terms of giving space to working women?
19. What advise do you have for any woman interested in taking on a leadership role in Pakistan?

**Semi- structured Protocol for Family Members of Women in Leadership Positions**

1. Tell me about yourself. How was growing up like?
2. What were the views about working women when you were young?
3. What were the views about working women when you were studying?
4. What are the views about Working Women nowadays?
5. What is your opinion when you see working women nowadays?
6. What’s your opinion about women play any role in progression of a country?
7. What does is look like for women today as they are trying to achieve work life balance?
  a. Think about a woman in your family who has a job working outside the home. What is your relationship like with her?
  b. Has her work ever impacted your relationship?
8. In your opinion, do you think women have any influence on the community?
9. Do you think women holding leadership positions can contribute to Pakistani society? Why or why not?
